# An Ontology-Based Reasoning Framework for Querying Satellite Images for Disaster Monitoring

**DOI:** 10.3390/s17112545

**Published:** 2017-11-05

**Authors:** Marjan Alirezaie, Andrey Kiselev, Martin Längkvist, Franziska Klügl, Amy Loutfi

**Affiliations:** Center for Applied Autonomous Sensor Systems, Örebro University, 702 81 Örebro, Sweden; andrey.kiselev@oru.se (A.K.); martin.langkvist@oru.se (M.L.); Franziska.Klugl@oru.se (F.K.); amy.loutfi@oru.se (A.L.)

**Keywords:** satellite imagery data, natural hazards, ontology, reasoning, path finding

## Abstract

This paper presents a framework in which satellite images are classified and augmented with additional semantic information to enable queries about what can be found on the map at a particular location, but also about paths that can be taken. This is achieved by a reasoning framework based on qualitative spatial reasoning that is able to find answers to high level queries that may vary on the current situation. This framework called SemCityMap, provides the full pipeline from enriching the raw image data with rudimentary labels to the integration of a knowledge representation and reasoning methods to user interfaces for high level querying. To illustrate the utility of SemCityMap in a disaster scenario, we use an urban environment—central Stockholm—in combination with a flood simulation. We show that the system provides useful answers to high-level queries also with respect to the current flood status. Examples of such queries concern path planning for vehicles or retrieval of safe regions such as “find all regions close to schools and far from the flooded area”. The particular advantage of our approach lies in the fact that ontological information and reasoning is explicitly integrated so that queries can be formulated in a natural way using concepts on appropriate level of abstraction, including additional constraints.

## 1. Introduction

Reliable and automated satellite image classification is of increasing importance for disaster management and climate change monitoring. In addition to the recognition of objects and entities in satellite images, it is also important to be able to reason about these entities and eventually answer queries posed by human operators in order to guide decision making processes.

An important enabler for automated reasoning and also for human machine interaction is that the images contain semantic annotations upon which intelligent and automated retrieval or planning processes can rely. These semantic annotations are typically based on pre-defined concepts about objects and entities. They should also contain rich domain knowledge and information about relations between the concepts in order to facilitate more complex and elaborate reasoning. Examples of such reasoning could be the reasoning about spatial and temporal information between and about objects. In natural disaster situations, e.g., a flood, the duration of rescue activities, such as finding safe areas, or navigation-related tasks, can be considerably reduced by enabling machines to do such automated reasoning processes.

This paper presents a complete framework, SemCityMap, which takes as input large scale satellite images and automatically extracts and enriches semantic annotations to facilitate a variety of tasks such as retrieving regions with specific (e.g., spatial) features, or finding paths between two specific areas. The city that we used for illustrating the features of the framework is Stockholm. According to the recent studies of climate researchers [1], many different parts of Stockholm are under risk for floods. This is mainly because of an increase in the average temperature which causes more rain in autumn and winter. Thus, the scenario of a flooded Stockholm is actually realistic. Therefore, we have defined a disaster scenario in which the central part of Stockholm is mostly covered by water.

In the proposed framework, first, a convolutional neural network (CNN) is used to provide rapid classification of entities in the map using pre-defined rudimentary categories (roads, building, trees etc.). The map with these initial labeling is augmented with additional information from publicly available sources in order to extract addresses, affordances, and other high-level knowledge. By organizing this information in ontologies, the reasoning framework exploits the combination of explicitly defined domain knowledge in form of ontology patterns with up-to-date situational information extracted from the satellite images. Thus, the overall system is able to answer high-level queries about the current status and situation in the map. Due to the ontological reasoning involved, queries can contain concepts that do not directly correspond to original labels, as well as involve complex combinations of additional conditions. They may include even features of the situation before the disaster.

The main contributions of this work are:

1. We propose a principled approach to semantically enrich annotations in a map by geometrically aligning classification output from a CNN to publicly available geo-data.
2. Instead of building our own ontology from scratch, we show how to reuse publicly available geo-related ontologies in order to represent geometry, spatial relations and affordances of entities. This extension is called OntoCity.
3. We demonstrate the framework, SemCityMap, for path finding tasks in a flood scenario where high-level semantics are integrated into the path finding algorithm per se.
4. We evaluate query performance under different conditions formulated in the queries. These conditions involve spatial relations, connectivity, and the classification of the entity in the map (buildings, roads, schools, etc.).

Before presenting related work, we briefly explain what it actually means for the SemCityMap framework to be based on ontological reasoning.

### 1.1. Ontology-Based Reasoning

In both the fields of Geography Information System (GIS) and Remote Sensing (RS), ontologies are being increasingly used as a semantic representational model [2]. Semantic models in general define a domain of discourse in the form of relevant concepts along with their relations and constraints [3]. Such an ontological representation can help to reduce the semantic gap between different abstraction levels of data [4], that in case of GIS can range from the digital satellite imagery data of a scene under process, to the symbolic information in the form of objects or physical entities in the scene.

Ontological relations which are in the form of logic-based axioms are used by reasoning methods in order to infer both implicit and explicit information. For instance, with a user-given a concept, an ontology-based reasoning process understanding the ontology languages is able to retrieve more general/specific concepts using the subsumption relations between concepts. Relations between concepts can be more complicated and specify further features (e.g., in the form of logical constraints) about the concept by relating it to the others. For instance, let us assume that an ontology designed to represent geo-related concepts contains concepts such as *river*, *building*, *road*, *bridge*, etc. The concept *bridge* can be more (e.g., geometrically) related to the concept *river* as bridges may be built to cross rivers, connecting two shores. If such relation along with further geometrical/spatial or topological information (e.g., defining what is a “shore”) is added to the ontology, a reasoner can infer for which instances on the map the relation holds and thus identify an object as a bridge. This provides not just a high-level language to talk about what can be found on a map, but enables to add common-sense knowledge about the current situation. Such explicit definition of concepts and relations supports reusability of ontological information.

Ontological reasoning whose performance mainly relies on the proper definition of concepts and their relations can benefit from the general reusability of ontologies and fetch further information by going beyond a specific domain. Reusability of ontologies is being studied in the ontology design patterns community [5] whose concern is about designing ontological modules generic enough to be used in multiple domains. In this paper, we will explain how OntoCity as the main representation model in our work is using ontology patterns to assist the ontology reasoner in retrieving useful information for the user.

### 1.2. Related Works

Due to their specific features such as knowledge reusability that leads to semantic interoperability, ontologies are well contributing in representation of different domains [6]. For instance, in the GIS literature, ontologies are used for different purposes such as image classification, information retrieval and decision making processes. In image classification, as an inevitable process in geo-related applications using satellite imagery data, ontologies are used to semantically relate the imagery data to relevant objects in order to improve the classification results. The work presented in [7] proposes a knowledge-driven methodology to enhance the object-based semantic classification method using ontologies that express objects, their size and spatial relations. Likewise, in [8] an ontology-based solution is suggested to classify ocean satellite images using an ontology which provides high and low level features of regions extracted from an ocean scene. Besides the ontology languages such as RDF (Resource Description Framework) and OWL (Web Ontology Language), ontologies allow us to use further constraints in definition of concepts, understandable by reasoners that can contribute in retrieving further (implicit) information about a given domain. For instance the approach explained in [9] is able to retrieve geographic information using a qualitative representation where the spatial and directional relations are defined in a way understandable by qualitative reasoner.

Apart from the role of ontologies in improving the analysis of geo-related data shown in the aforementioned research work, we recently see an increase in use of ontologies for more specific applications such as emergency management including rescuing and disaster relief. There has been considerable amount of work based on ontologies in the area of natural disasters. This is due to the ontologies’ semantic interoperability feature that can increase the chance of retrieving required information. For instance, [10] proposes an ontology model to represent events along with the actions required to be taken in specific emergency situations. Another work presented in [11] introduces an ontology which is developed based on SUMO (Suggested Upper Merged Ontology) in order to model an emergency rescue patterns automatically generated based on the given context. In particular, the OntoFire ontology explained in [12] is part of a portal that is focused on wildfires, and offers different services such as navigation automatically generated based on the relations discovered between the given data. Moreover, FloodOntology introduced in [13] is used for flood forecasting and is composed of entities related to the measurement elements such as hydrological/hydraulic parameters used to predict the time and the duration of the floods.

In all the aforementioned work, sharing different types of data and then reasoning upon it was the key, for which semantic interoperability is a necessity [14]. Semantic interoperability is better achievable if the designing process of ontologies respects some generic ontology patterns that are usually relying on upper level ontologies [5]. However, most of the aforementioned ontologies used in disaster scenarios are designed in an ad-hoc way and are not based on upper level ontologies.

In this paper, we focus on (re)using existing geo-ontologies as one step towards enabling semantic interoperability in geo-related domains. The ontology proposed in this work is called OntoCity as an extension of GeoSPARQL ontology suggested by the Open GeoSpatial Consortium (OGC). We will explain how this extension enables us to reason using semantically enriched data.

We have so far explained a brief introduction to the ontological reasoning in Section 1.1, and also its role in the GIS literature in Section 1.2. Since the main focus of the paper is representing the role of OntoCity in querying satellite data, we continue by explaining the basis of OntoCity, as the representational model in the SemCityMap framework, in terms of its building blocks in Section 2. In order to show how OntoCity is instantiated, in Section 3, we provide the link between the data layer including data acquisition and data processing and the ontology population process in. Given the populated ontology, we are ready to go through the reasoning process in Section 4, where we explain which types of queries are needed to be answered. In Section 5, we visually explain the pipeline of SemCityMap. This section also provides us enough room to briefly introduce our graphical user interface which although did not have a main role in the representation and the reasoning part, however, is quite important to visualize the outputs of the reasoner. Section 6 talks about the implementation of a disaster scenario and measure the computational features of the framework such as the searching time of the queries applied upon real data of Stockholm. We then conclude the paper in Section 7 which is followed by a brief discussion on the future work in Section 8.

## 2. Modeling in Ontologies

In order to enable intelligent reasoning upon imagery data for disaster management, a representation model whereby the data along with its relations are represented in a higher abstraction (i.e., symbolic) level, is required. For this, we have developed an ontology called OntoCity. OntoCity is constructed based on GeoSPARQL proposed by OGC as a standard vocabulary for geospatial data in RDF that enables qualitative spatial reasoning upon this type of data [15]. OntoCity is designed to represent all the structural aspects of a city including the building blocks, their types (e.g., natural or man-made), and their relations (e.g., spatial relations, affordances, etc.), which might be used for disaster relief.

In the following subsections we describe the structure of GeoSPARQL and then its extension formed in OntoCity. Since the definitions of axioms in the ontology are given in Description Logic (DL) language, we first briefly explain the notations used in the following sections (for further details, we refer the readers unfamiliar with the DL syntax to the Basic DL tutorial in [16]):

- The expression “name1:name2” refers to the entity name2, which belongs to the ontology name1.
- The **subsumption** relation shown as “A⊑B” means concept **A** is a subset (a specialization) of concept **B**.
- The **full existential quantification** shown as “∃R.C” indicates all the concepts whose instances have at least one relation with concept **C** via property **R**.
- The **number restriction** shown as “= n **R**”, where n is a numerical value, indicates an ontological concept whose instances are related to other concepts/values n-times via the property **R**.

### 2.1. GeoSPARQL

GeoSPARQL provides a generic basis to define any spatial object (as an instance of the class geos:SpatialObject (The prefix geos refers to the name of the GeoSPARQL ontology.)) that has a geometry in the physical world. The class that is responsible to represent such entities in geo-related domain is called geos:Feature which is subsumed by the class geos:SpatialObject (geos:Feature ⊑ geos:SpatialObject). Each instance of the class feature is connected to another instance belonging to the class geos:Geometry via the property geos:hasGeometry as follows:

(1) $$\begin{array}{cc} \mathrm{geos}:\mathrm{Feature}\;⊑\; & \exists \;\mathrm{geos}:\mathrm{hasGeometry}.\mathrm{geos}:\mathrm{Geometry} \end{array}$$

Using the property geos:asWKT, each geometry indicates all the boundaries (inner and outer) of the object in the form of the WKT (Well Known Text) string format which refers to a literal (a rdfs literal) value specifying a list of coordinates defining the boundary:

(2) $$\begin{array}{cc} \mathrm{geos}:\mathrm{Geometry}\;⊑\; & \exists \;\mathrm{geos}:\mathrm{asWKT}.\mathrm{rdfs}:\mathrm{Literal} \end{array}$$

The class geos:Geometry as such has its own taxonomy to define specialized geometries such as polygons, rectangles, etc., as its subclasses.

**Figure 1:**
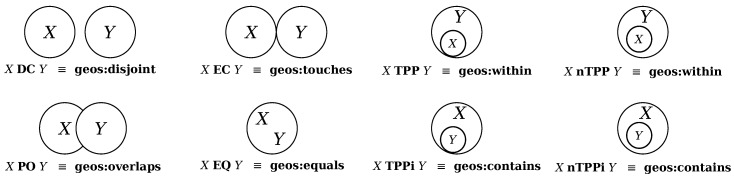
RCC-8 spatial relations defined in GeoSPARQL, where DC = disconnected, EC = externally connected, TPP = tangential proper part, nTTP = non-tangential proper part, PO = partially overlapping, EQ = equal, TPPi = tangential proper part inverse, nTTPi = non-tangential proper part inverse.

Furthermore, GeoSPARQL provides a set of properties that qualitatively represent the spatial relations defined in RCC-8 (Region Connection Calculus) [17]. According to the RCC-8 calculus, any spatial relation between any two spatial objects can be represented in one of the 8 basic RCC relations shown in Figure 1.

In next section, we explain how OntoCity is built by extending GeoSPARQL.

### 2.2. DUL Ontology

In construction of OntoCity we have also used the upper ontology DOLCE+DnS Ultralite (www.ontologydesignpatterns.org/ont/dul/DUL.owl.) (DUL). By upper ontology we refer to an ontology containing general concepts and relations that are publicly used across all domains [18]. As we see in next section, the reason behind using DUL in OntoCity is to represent events in the form of instances of the class DUL:Event.

### 2.3. OntoCity

The OntoCity ontology is an extension of GeoSPARQL. This extension has been done in three aspects including (I) refactoring of the spatial relations, (II) representing a taxonomy of the geos:Feature class, (III) defining path connectivity, that are separately explained in the following sections.

#### 2.3.1. Refactoring of Spatial Relations

As shown in Figure 1, there are several distinct relations in RCC-8 that all are indicating intersection relations between two given objects (e.g., goes:overlaps, geos:within, geos:contains). Although there are differences between the aforementioned relations that differentiate them from each other, however, in many situations, we are only interested to know if the two objects (or their bounding boxes) are intersecting or not. For the sake of practicality, we have defined a new class in OntoCity called ontocity:intersects that as a superclass subsumes the relations geos:overlaps, geos:contains and geos:within. In other words, whenever we use the ontocity:intersects relation between any pair of spatial objects we mean that at least the boundary boxes of these two objects are sharing a common space.

Furthermore, in OntoCity, we have defined a new object property, called ontocity:hasSpatialRelation, as the superclass of all the relations in RCC-8 calculus. The new property is used to complete the definition of geos:Feature in the sense that each instance of this class has at least one spatial relation with another feature.

(3) $$\begin{array}{cc} \mathrm{geos}:\mathrm{Feature}\;⊑\; & \exists \;\mathrm{ontocity}:\mathrm{hasSpatialRelation}.\mathrm{geos}:\mathrm{Feature} \end{array}$$

#### 2.3.2. Taxonomy of geos:Feature

The ontocity:hasSpatialRelation property is used in the definition of the subclasses of the geos:Feature class. As said in Section 2.1, a feature in GeoSPARQL represents any object that has a geometry. In OntoCity, we have defined 3 direct subclasses of the class geos:Feature including ontocity:Region, ontocity:Segment and ontocity:Area.

The class ontocity:Region as a direct subclass of the class geos:Feature is used to represent all the regions or structures in a city that have a label (e.g., building, river, parking place, etc.). The ontocity:Region class is as such categorized into 2 main subclasses ontocity:ManmadeRegion and ontocity:NaturalRegion.

(4) $$\begin{array}{cc} \mathrm{ontocity}:\mathrm{Region}\;⊑\; & \;\mathrm{geos}:\mathrm{Feature}\;⊓\\ & \;\exists \;\mathrm{ontocity}:\mathrm{hasSpatialRelation}.\mathrm{ontocity}:\mathrm{Region} \end{array}$$

(5) $$\begin{array}{cc} \mathrm{ontocity}:\mathrm{ManmadeRegion}\;⊑\; & \;\mathrm{ontocity}:\mathrm{Region} \end{array}$$

(6) $$\begin{array}{cc} \mathrm{ontocity}:\mathrm{NaturalRegion}\;⊑\; & \;\mathrm{ontocity}:\mathrm{Region} \end{array}$$

The categorization of the class region is also formed based on the other properties such as the land cover of regions (e.g., water area, or vegetation area taken from the classified satellite data) or their affordances (e.g., a region can be walked on as part of a path). The following example subsumption relations show the direct subclasses of the class ontocity:Region:

(7) $$\begin{array}{cc} \mathrm{ontocity}:\mathrm{PavedArea}\; & ⊑\;\;\mathrm{ontocity}:\mathrm{GroundArea}\;⊑\;\;\mathrm{ontocity}:\mathrm{Region} \end{array}$$

(8) $$\begin{array}{cc} \mathrm{ontocity}:\mathrm{VegetationArea}\; & ⊑\;\;\mathrm{ontocity}:\mathrm{Region} \end{array}$$

(9) $$\begin{array}{cc} \mathrm{ontocity}:\mathrm{WaterArea}\; & ⊑\;\;\mathrm{ontocity}:\mathrm{Way}\;⊑\;\;\mathrm{ontocity}:\mathrm{Region} \end{array}$$

**Figure 2:**
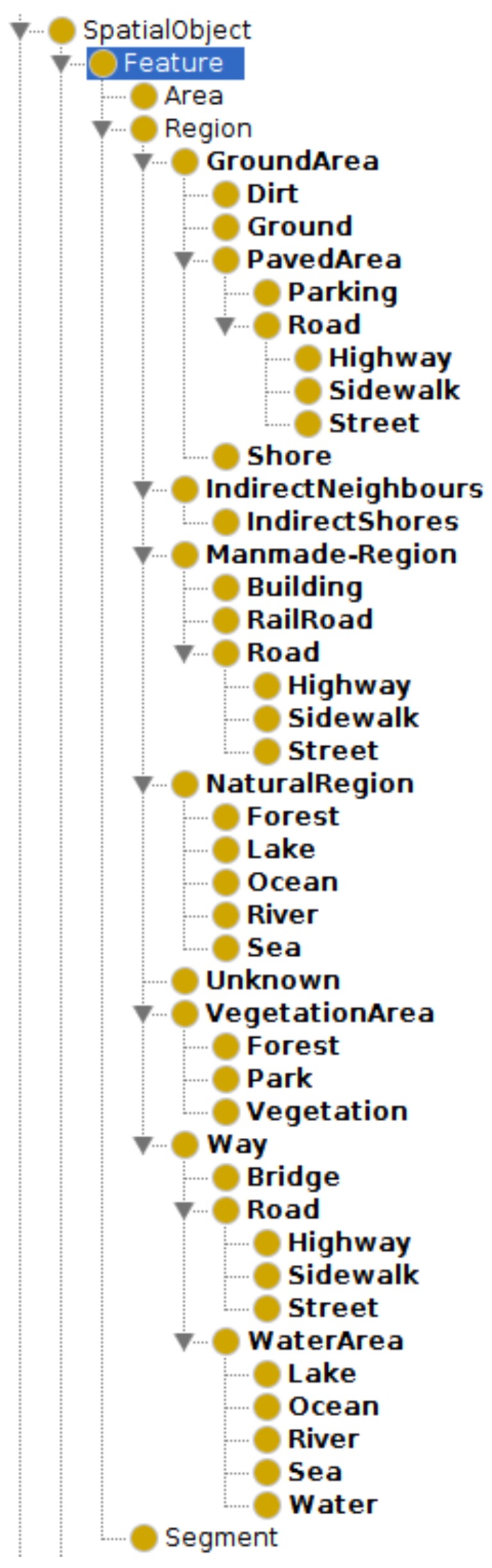
Hierarchy of the subclasses of the class geos:Feature in OntoCity.

Each of the direct subclasses of the class ontocity:Region has its own subclasses as either a man-made or natural region types (see Figure 2). For instance, as shown in the following, the concept ontocity:Road indicates a region on the scene that is man-made and can be used as a way (i.e., a route) in a city. Likewise, a river is also a region, but natural one which is used as a way (for example for boats) with water texture:

(10) $$\begin{array}{cc} \mathrm{ontocity}:\mathrm{Road}\;⊑\; & \;\mathrm{ontocity}:\mathrm{ManmadeRegion}\;⊓\;\mathrm{ontocity}:\mathrm{PavedArea}\;⊓\;\mathrm{ontocity}:\mathrm{Way} \end{array}$$

(11) $$\begin{array}{cc} \mathrm{ontocity}:\mathrm{River}\;⊑\; & \;\mathrm{ontocity}:\mathrm{NaturalRegion}\;⊓\;\mathrm{ontocity}:\mathrm{WaterArea}\;⊓\;\mathrm{ontocity}:\mathrm{Way} \end{array}$$

As we will show in Section 3.3, all the instances of the class geos:Feature, regardless of their types, are represented in the form of their boundaries and also the spatial relations between them. For being used in disaster management, their semantics can be further enriched. Most of the rescue tasks in emergency situations in outdoor environments are based on navigation and way finding in destroyed environments. Considering the semantics of regions (i.e., the affordance which shows what they can be used for) and consequently their alternatives (in case of obstruction or danger) can be an essential help in automatic navigation (or path finding) processes. In the following subsection, we explain another extension in OntoCity which is about defining paths along with their alternatives.

Apart from the class ontocity:Region which represents all the physical geo-entities on the ground, there are two other direct subclasses of the geos:Feature class called ontocity:Segment and ontocity:Area. The main feature of these two classes is that they are, unlike the class ontocity:Region, representing more abstract spatial concepts that are not physically existing on the ground or not labeled with a specific term referring to a cover land.

An instance of the class ontocity:Segment is defined as a rectangular feature on the map and holds (intersects with) several regions (see Axiom (12)). As we will explain in Section 3.3, instances of the class ontocity:Segment are used to improve the performance of the query system.

(12) $$\begin{array}{cc} \mathrm{ontocity}:\mathrm{Segment}\;⊑\; & \mathrm{geos}:\mathrm{Feature}\;⊓\\ & \;\exists \;\mathrm{geosp}:\mathrm{hasGeometry}.\mathrm{geos}:\mathrm{Rectangle}\;⊓\\ & \;\exists \;\mathrm{ontocity}:\mathrm{intersects}.\mathrm{ontocity}:\mathrm{Region} \end{array}$$

Likewise, each instance of the class ontocity:Area represents an area in the form of a polygon (and not necessarily a rectangle) in which an event (either natural or man-made) has occurred. By event we refer to the class DUL:Event. As we will see in Section 3.3, the geometry of the instances of the class ontocity:Area is considered by the query system when it is assumed to query about locations impacted by an event.

(13) $$\begin{array}{cc} \mathrm{ontocity}:\mathrm{Area}\;⊑\; & \mathrm{geos}:\mathrm{Feature}\;⊓\\ & \;\exists \;\mathrm{ontocity}:\mathrm{hasEvent}.\mathrm{DUL}:\mathrm{Event}\;⊓\\ & \;\exists \;\mathrm{ontocity}:\mathrm{intersects}.\mathrm{ontocity}:\mathrm{Region} \end{array}$$

As Axiom (13) shows, the spatial relation defined between an area and a region is the relation ontocity:intersects. It means that an area involved in an event can intersect with some parts of a region (and not necessarily contains the whole region).

### 2.4. Path Connectivity in OntoCity

A region can also represent a way (i.e., a transportation route) located between two other regions. For this, we have defined another class, ontocity:Way ⊑ ontocity:Region, which subsumes the classes ontocity:Road (and subsequently ontocity:Street, ontocity:HighWay, etc.) and ontocity:River. To complete the definition of the class ontocity:Way an extension is required. This extension involves the representation of a way (i.e., path) along with the regions connected through it. Representation of a way with its involved regions asks for a relation that is defined between more than two elements. More specifically, a path is seen as an n-ary relation semantics that can be modeled via the generic n-ary ontology pattern [19].

In OntoCity, the n-ary path relation needs to be symmetric (a symmetric n-ary or s-n-ary). According to [20], assuming s-n-ary as a function upon the two given argument x and y, the following relation holds:

*s-n-ary(x,y) = z* ⇔ *s-n-ary(y,x) = z*.

**Figure 3:**
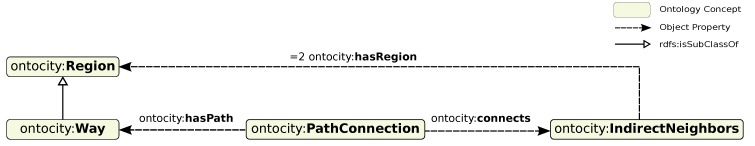
The PathConnection Pattern in OntoCity representing a symmetric n-ary relation.

More specifically, our definition in path connectivity (symmetric n-ary relation) in OntoCity represented in Figure 3, relies on the fact that: region x is connected to the region y via the path z, if and only if the region y is also connected to region x via the same path. As Figure 3 shows, there are two extra classes in OntoCity, ontocity:IndirectNeighbors and ontocity:PathConnection, where the former represents the two regions in OntoCity which are connected via a path, and the latter indicates the path (as an instance of ontocity:Way) connecting these two regions together. The DL representation of the classes are as follows:

(14) $$\begin{array}{cc} \mathrm{ontocity}.\mathrm{IndirectNeighbors}\;⊑\; & =\;2\;\mathrm{ontocity}.\mathrm{hasRegion}.\mathrm{ontocity}:\mathrm{Region} \end{array}$$

(15) $$\begin{array}{cc} \mathrm{ontocity}.\mathrm{PathConnection}\;⊑\; & \exists \;\mathrm{ontocity}.\mathrm{connects}.\mathrm{ontocity}.\mathrm{IndirectNeighbors}\;⊓\\ & =\;1\;\mathrm{ontocity}.\mathrm{hasPath}.\mathrm{ontocity}:\mathrm{Way} \end{array}$$

The relations between concepts are provided via three properties, ontocity:hasPath, ontocity:connects and ontocity:hasRegion.

**Figure 4:**
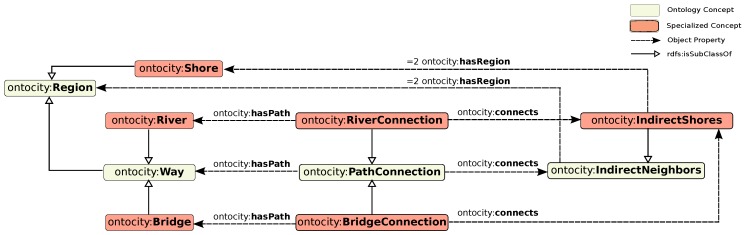
Extension of the PathConnection in OntoCity - River and Bridge example.

By specializing the aforementioned classes, OntoCity contains different types of paths. For instance, as shown in Figure 4, the class ontocity:PathConnection subsumes the class ontocity:RiverConnection whose path and region parts are defined as subclasses of the class ontocity:River ⊑ontocity:Way and the class ontocity:Shore⊑ ontocity:Region, respectively:

(16) $$\begin{array}{cc} \mathrm{ontocity}:\mathrm{RiverConnection}⊑\; & \mathrm{ontocity}:\mathrm{PathConnection}\;⊓\\ & \exists \;\mathrm{ontocity}:\mathrm{connects}.\mathrm{ontocity}:\mathrm{IndirectShores}\;⊓\\ & =\;1\;\mathrm{ontocity}:\mathrm{hasPath}.\mathrm{ontocity}:\mathrm{River} \end{array}$$

(17) $$\begin{array}{cc} where:\;\mathrm{ontocity}:\mathrm{IndirectShores}⊑\; & \mathrm{ontocity}:\mathrm{IndirectNeighbors}\;⊓\\ & =\;2\;\mathrm{ontocity}:\mathrm{hasRegion}.\mathrm{ontocity}:\mathrm{Shore} \end{array}$$

(18) $$\begin{array}{cc} and,\;\mathrm{ontocity}:\mathrm{Shore}⊑\; & \mathrm{ontocity}:\mathrm{GroundArea}\;⊑\;\mathrm{ontocity}:\mathrm{Region} \end{array}$$

Likewise, the class ontocity:PathConnection is specialized to the class ontocity:BridgeConnection which defines bridges as paths over water areas as follows:

(19) $$\begin{array}{cc} \mathrm{ontocity}:\mathrm{BridgeConnection}⊑\; & \mathrm{ontocity}:\mathrm{PathConnection}\;⊓\\ & \exists \;\mathrm{ontocity}:\mathrm{connects}.\mathrm{ontocity}:\mathrm{IndirectShores}\;⊓\\ & =\;1\;\mathrm{ontocity}:\mathrm{hasPath}.\mathrm{ontocity}:\mathrm{Bridge} \end{array}$$

(20) $$\begin{array}{cc} where:\;\mathrm{ontocity}:\mathrm{Bridge}⊑\; & \mathrm{ontocity}:\mathrm{Way}\;⊑\;\mathrm{ontocity}:\mathrm{Region} \end{array}$$

The subclasses of the class ontocity:PathConnection can be further related to each other if there are similarities in types of *regions* or *ways* mentioned in their definitions. For example, the two classes ontocity:BridgeConnection and ontocity:RiverConnection are similar in the sense that both are able to connect shore areas. Finding these similarities might help a path finding process to find alternative paths for those located in forbidden or unreachable areas in certain situations (e.g., dangerous areas such as rivers in case of flood).

In Section 6 we will show how OntoCity is populated with instances of the path connectivity pattern. For now, let us assume that OntoCity is populated with all the regions and their connections.

Given a specific region type C as a constraint (e.g., a forbidden area) which is also defined in OntoCity (C⊑ontocity:Region), by running the Query (21), we will be able to retrieve all the alternatives for the region type C. By alternatives, we mean those regions (more specifically instances of the class ontocity:Way) that are although different from C (and therefore legal to be part of a path), they are similar to C in the sense that they play the same role as C in terms of connecting specific region types. Using the path connectivity pattern in OntoCity and the query, we can automatically retrieve paths compatible with the situation.

(21) $$\begin{array}{cc} AlternativeWays\;=\;\{\;r_{i}\in R_{i}\;|\; & R_{i}\;⊑\;\mathrm{ontocity}:\mathrm{Way}\;\wedge \;r_{i}\;\notin \;C\;\wedge \;\exists \;c\;\in \;C\;\wedge \\ & \;\exists \;p_{1}\;\in \;P_{1}\;⊑\mathrm{ontocity}:\mathrm{PathConnection}\;\wedge \;\exists \;p_{2}\;\in \;P_{2}\;⊑\mathrm{ontocity}:\mathrm{PathConnection}\;\wedge \\ & \;\exists \;n\;\in \;N\;⊑\mathrm{ontocity}:\mathrm{IndirectNeighbors}\;\wedge \\ & \left(c,p_{1}\right)\;\in \;\mathrm{ontocity}:\mathrm{hasPath}\;\wedge \;\left(p_{1},n\right)\;\in \;\mathrm{ontocity}:\mathrm{connects}\;\wedge \\ & \left(r_{i},p_{2}\right)\;\in \;\mathrm{ontocity}:\mathrm{hasPath}\;\wedge \;\left(p_{2},n\right)\;\in \;\mathrm{ontocity}:\mathrm{connects}\;\} \end{array}$$

For instance, assuming the situation does not allow us to cross water areas, we can specialize the Query (21) by assuming C=ontocity:WaterArea which will be equivalent to the extension of the path connectivity shown in Figure 4. According to this specialized query, the reasoner will return back an alternative for water areas, which can however be still used to connect shore areas (e.g., bridges as alternatives for rivers).

## 3. Populating OntoCity for a Particular Disaster

In order to clarify how OntoCity is used, and how the system is able to make sense of the raw satellite imagery data, we briefly go to the details of the data acquisition and the classification processes. After that, the instantiation of the ontology along with the reasoning process will be explained.

### 3.1. Data Acquisition

In the presented system, multiband satellite imagery data is used both for classification and for visualization. The data is represented in two forms: orthorectified images and reconstructed 3D meshes. Ortho images are used for classification. The dataset consists of 7 primary and 7 synthetic bands. The resolution of the image data is 0.5 m/px. The images are orthorectified and referenced to actual GIS data. The summary of the color bands is given in Table 1.

For visualization, the stereophotogrammetrically reconstructed 3D mesh data is used to provide a user a possibility to inspect the area from different viewpoints.

### 3.2. Classification and Segmentation

The classification of the map is performed on a per-pixel level using a Convolutional Neural Network (CNN) [21] based on 7 rudimentary categories of labels including (vegetation, ground, road, building, water, railroad and parking). The procedure and the structure of the network follows the work by [22]. The surrounding area of the pixel to be classified is used as input to the CNN. A softmax layer is attached to the output of the CNN for the final classification. In this work, a one-layer CNN is used on a 25×25 input patch with 50 filters of filter size 11 and pooling dimension 5. These hyperparameters were selected to get an output of 3×3 from the CNN. The softmax layer was set to 500 hidden units. The whole network is trained end-to-end with supervised backpropagation on the training set until the accuracy on the validation set has not been improved in the last 10 epochs. The training/validation/test sets are obtained by randomly extracting 1000 input patches of size 25×25 from each of the 7 classes and then dividing them into a 80/10/10 split. The classification result on the test set is presented in Section 6.2.1.

**Figure 5:**
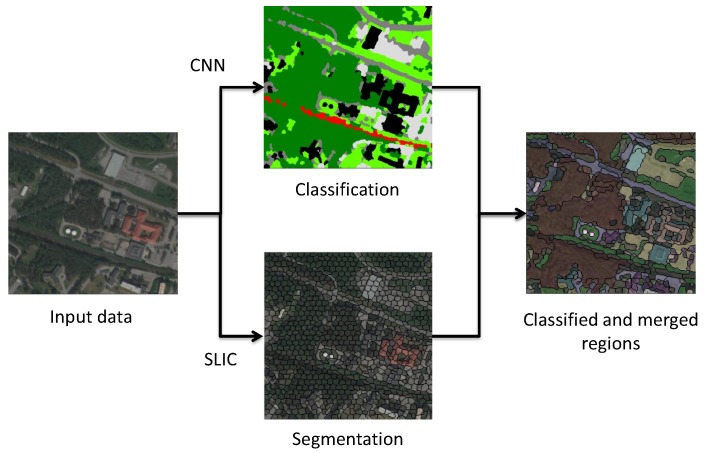
Merged classified regions are obtained by averaging the classification results (Black = building, Dark Green = Vegetation, Light Green = Ground, Gray = Road, Red: RailRoad) over all classified pixels in each region from an initial SLIC segmentation. Regions are merged if the average classification certainties for two connecting regions are both above a certain threshold.

After the whole map has been classified, the segmented (Segmentation here refers to the SLIC segmentation algorithm and is different from the instances of the class onto:Segment defined in the ontology.) regions that are used for later are obtained by using the SLIC segmentation algorithm [23] and then merged using the pixel classifications, see Figure 5. The purpose of merging regions is to reduce the number of regions and to get regions that better represents real world areas/objects.

### 3.3. Population of OntoCity

Instantiation of classes in OntoCity is in two steps of region instantiation (related to the classes ontocity:Region and ontocity:Segment) and event instantiation (related to the classes ontocity:Region, ontocity:Area and DUL:Event) explained in the following subsections.

#### 3.3.1. Region Instantiation

Region instantiation process includes three different steps including regions’ boundary extraction from the classification output, generating instances of the class ontocity:Segment (i.e., segmentation (Segmentation in ontology refers to the class onto:Segment and is different from segments generated by the classifier.) of the map) and representing spatial relations between regions in a segment.

Each generated segment (which is henceforth referred to as region) is represented as an instance of a subclass of ontocity:Region, equivalent to its label (i.e., one of the rudimentary labels mentioned in Section 3.2. Assuming $L_{R}$ indicates a preliminary label assigned by the classifier to region *r*${}_{i}$, and refers to the region type *R* (e.g., *Building*, *Parking*, etc.,) the first step of populating OntoCity is as follows:

(22) $$\begin{array}{c} r_{i}\in R\;where:\;R\;⊑\;\mathrm{ontocity}:\mathrm{Region} \end{array}$$

**Figure 6:**
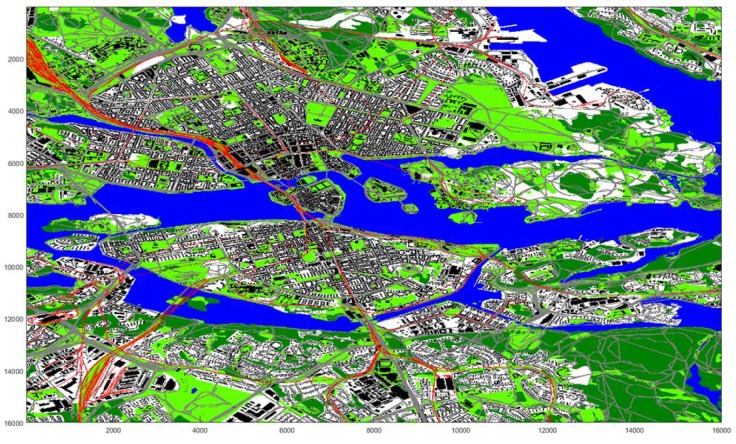
Labels provided by Lantmäteriet.

OntoCity also includes the geometry of each region. Given the boundaries of a region, we are able to enrich the (preliminary) label ($L_{R}$) of the region assigned by the classifier. As shown in Figure 2, the hierarchy of regions in OntoCity are more than the 7 rudimentary labels (e.g., building, water, etc.) used by the classifier. Thanks to the availability of geo data on publicly available sources such as OpenStreetMap (OSM) [24] or the map provided by Lantmäteriet (a Swedish government body responsible for mapping the country), we could automatically extract more specific labels for the classified regions. Given the region *r*${}_{i}$, a new label could be retrieved from the sources assigned to the area located at the same place that the region’s boundary indicates. The labels provided by Lantmäteriet is shown in Figure 6.

Let us assume that $L_{R}$ is the label of region *r*${}_{i}$ set by the classifier, and $L_{C}$ is the candidate label for the region given by its counter part on public sources. The label enrichment process updates OntoCity w.r.t the following condition:

(23) $$\begin{array}{c} r_{i}\in R\;\wedge \;C⊑R\;\Rightarrow \;r_{i}\in C \end{array}$$

In other words, the instance *r${}_{i}$* will also be the instance of the class *C* (relevant to the candidate label $L_{C}$) if the class *C* in OntoCity is subsumed by the original class label *R*. For instance, if a region (*r${}_{i}$*) is labeled as *Building* ($L_{R}$ = *Building*) by the classifier (where: ontocity:Building⊑ontocity:Region), and at the same time, the enrichment process finds from other sources a region at the same location as *r${}_{i}$*, labeled as *School* ($L_{C}$ = *School*), then OntoCity will be updated by a new instantiation as *r${}_{i}$*∈ontocity:School only if ontocity:School⊑ ontocity:Building. Details of the label enrichment process based on public maps can be found in [25].

One of the main purpose behind representing the geometry of regions on the map is to enable the map to answer queries about the topology and the neighborhood of regions. As we will see in Section 4.1, there is a direct relation between the time these queries take to answer and the number of regions modeled in the ontology. In order to improve the scalability of the query system in terms of the number of regions, we have considered another concept in OntoCity called ontocity:Segment ⊑geos:Feature, that as said in Section 2.3, represents rectangular spatial objects. Each segment instance in OntoCity is supposed to cover a particular part of the map holding a number of regions. Depending on the size of the map and also its resolution, we divide the map to a number of pairwise disjoint rectangular segments that altogether cover the entire map. Each time a region *r${}_{i}$* is extracted from the classification output, we populate the ontology also with all the segments (i.e., instances of the ontocity:Segment class) geometrically intersecting with *r${}_{i}$*. The intersection relation (ontocity:intersects) between a region and a segment can be in the form of either geos:contains or geos:overlaps in the RCC-8 calculus.

Finally, after generating the instances of the segment class in the ontology and representing the geometry of regions in each segment, we calculate the spatial relations between any pair of regions within a segment. For instance, if the two given regions *r*${}_{i}$ and *r${}_{j}$* belonging to one segment are direct neighbors, we add the axiom: “*r*${}_{i}$ geos:touches *r${}_{j}$*” in OntoCity saying that the two regions are directly connected to each other. Likewise, all pairs of regions belonging to disconnected segments are (implicitly) related to each other via the RCC-8 geos:disjoint relation.

#### 3.3.2. Event Instantiation

By event, we refer to any natural or man-made occurrence that happens at a certain time and place. Event as a concept has been already defined in plenty of ontologies mostly relying on the upper DOLCE Ultra Light (DUL) ontology, where we can find the modeled spatio-temporal aspect of an occurrence along with the participation of agents (e.g., humans) [26]. In OntoCity, we have so far considered only the spatial representation of events borrowed from the DUL ontology.

Representation of an event in OntoCity is in the form of an instance of the class DUL:Event which occurs at certain areas in a city. These areas are also indicated as instances of the class ontocity:Area subsumed by the class geos:Feature. Each area is likewise represented in the form of its boundaries that intersects one or several regions (see DL definition of the class ontocity:Area in Axiom 13).

The geometrical data of the flooded area in this work is generated using a rudimentary offline flooding simulator, which uses available DSM map to build a map of flooded areas. The flood simulation is performed in a static scenario, without taking into account any effects of the flood dynamics (such as flows and surface wetting). The flooding map generation is a multipass process, in which a set of flooding contours is generated for a given range of water levels with a given step. In own particular scenario, the lowest water level was set at 24 m above the zero level to 40 m, with a step of 0.5 m. On each step, the process starts with a binary thresholding of the DSM map at a given threshold (flooding level). This results in a binary bitmap, from which the contours of flooded areas are being extracted using Teh-Chin chain approximation algorithm [27]. Finally, after the contours are extracted, they are simplified using the Ramer–Douglas–Peucker algorithm (ϵ=5.0) [28,29] and filtered based on the area, so only the contours with the area of more than 750 m${}^{2}$ are used in the form of geometries for the instances of the class ontocity:Area.

## 4. Reasoning

In a disaster situation, a rescue team may need to query a semantically-enabled map for finding (safer) regions with specific (e.g., thematic or spatial) features, or for navigation services such as finding (collision free) paths or alternatives to specific areas, in case the main ones are inaccessible. For the rescue team requests, the system is assumed to consider certain semantic constraints given in the queries as well as in the definition of regions and relations asserted in the ontology. In other words, a reasoning process which understands the ontological axioms (such as the definition of concepts and constraints), is needed to be aligned with OntoCity.

In this paper, we consider two different types of queries including finding regions and finding paths that are further explained in the following subsections. For each type we also clarify the reasoning process applied upon OntoCity in order to answer the queries. Before going to the details it is worth mentioning that the reasoning process is done upon the populated ontology which contains the regions in the form of their geometries as well as their spatial relations between each other. The spatial relations are calculated using our geometrical processing module implemented in Java, as an off-line process. Given the populated ontology with all the regions and their links, we are able to run the our queries in the form of SPARQL as a pattern matching process language under the Jena framework [30].

### 4.1. Region Retrieval

By region retrieval we refer to a region searching process based on criteria such as types of regions along with the spatial metrics including directional relations, spatial relations and distances. More precisely, given a point $p_{c}$ chosen by the user on the map along with a set of criteria including the region types $R_{i}$ and $R_{j}$, an interval indicated by the lower bound $d_{l}$ and upper bound $d_{u}$, and also S as a binary spatial relation, a region retrieval process is defined as a searching process generating the answer set A defined as follows:

(24) $$\begin{array}{cc} A\;=\;\{\;r_{i}\in R_{i}\;|\; & R_{i}\;⊑\;\mathrm{ontocity}:\mathrm{Region}\;\wedge \\ & \;d_{l}\;\leq \;\mathrm{distance}\left(p_{c},\;r_{i}\right)\;\leq \;d_{u}\;\wedge \\ & ∄\;a\in \mathrm{ontocity}:\mathrm{Area}\;\wedge \\ & \;(r_{i},\;a)\in \mathrm{ontocity}:\mathrm{intersects}\;\wedge \\ & \;\exists \;r_{j}\in R_{j}\;⊑\;\mathrm{ontocity}:\mathrm{Region}\;\wedge \\ & \;(r_{i},\;r_{j})\in S\;⊑\;\mathrm{ontocity}:\mathrm{hasSpatialRelation}\;\} \end{array}$$

The answer set A contains all the regions of the given type $R_{i}$ as a subclass of the ontocity:Region class, located further than $d_{l}$ m and closer than $d_{u}$ meter to the given point $p_{c}$. Furthermore, according to the given query, these candidate regions are assumed not to be involved in some events. In other words, the query returns backs those regions that are not intersecting with an event-influenced parts of the city (i.e., refering to the instances of the class ontocity:Area). The query structure also has the capacity to mention some features in neighborhoods of the candidate regions. In other words, we can restrict the regions to only those that are in spatial relation S (as a subclass of the ontocity:hasSpatialRelation) with regions with specific type $R_{j}$.

For instance, the user can ask for all the *building*s ($R_{i}$ = ontocity:Building) which are located at a certain distance from point $p_{c}$ clicked on the map, and are connected (geos:touches) to *water area*s ($R_{j}$ = ontocity:WaterArea).

The time that the searching process requires to retrieve the regions of the answer set A, highly depends on the number of region instances in OntoCity. The more region instances are defined in OntoCity, the longer the search time is. In order to keep the system scalable (i.e., retaining an acceptable search time, regardless of the number of regions), the geometry of regions and segments are needed to be considered. In other words, instead of checking all the regions one by one and see if they are complying with the constraints given in A, we first retrieve the relevant segments which are the only ones supposed to contain the candidate regions.

**Figure 7:**
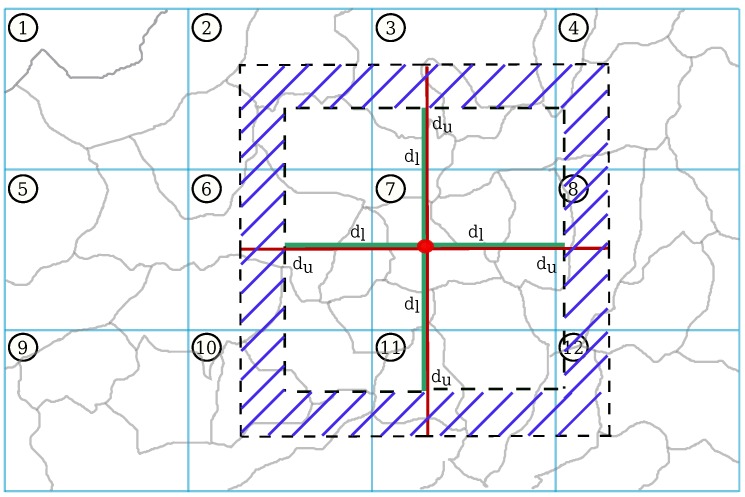
Retrieving segments relevant to the distance criteria given in the query. Segments are shown in blue rectangles. The red point indicates the point $p_{c}$ selected by the user on the map. The boundaries (lower and upper bounds) are also shown in green ($d_{l}$) and red ($d_{u}$) lines.

The main parameters indicating the relevant segments are the lower ($d_{l}$) and the upper ($d_{u}$) bounds given in the query. The relevant segments are those intersecting the rectangular border area calculated by subtracting the smaller square from the bigger one as shown in Figure 7 (the hatched area). The reason why we represented segments in the form of rectangles is to take advantage of the R-Tree [31] data structure used to more efficiently do spatial searching and calculate geometrical relations between regions and segments.

Depicted in the figure with dash lines, $S_{2d_{l}}$ and $S_{2d_{u}}$ are referring to the area of the smaller square (with the edge size of $2d_{l}$) and the bigger square (with the edge size of $2d_{u}$), respectively. Let us also name the hatched area as D which is defined as follows:

(25) $$\begin{array}{c} D\;=\;S_{2d_{u}}\;-\;S_{2d_{l}} \end{array}$$

As we can see, without taking the segments defined in the ontology into account, in order to retrieve the regions occupying some parts of D, the intersection of all the regions (in gray) with the area D has to be geometrically checked. However, instances of segments in OntoCity can be used to considerably reduce the number of regions in this geometrical process. For this to be possible, we first check the intersection of all the segments (12 instances in Figure 7) with the area D. It excludes the 4 segments 1, 5, 7, 9, as the irrelevant segments and only consider the regions in the relevant ones. This exclusion as we will show in Section 6 can highly influence the searching time of the query. It is also obvious to realize that if the size of the segments were smaller we could exclude even more segments in the beginning of the searching process.

### 4.2. Path Finding

After specifying features of regions appropriate to a specific situation, the system can be further asked to find a possible path from the given point $p_{c}$ to the found regions, which satisfy certain constraints based on the context or the environmental conditions. However, finding paths between a given point and a region is not always straightforward. Path finding known as the subproblem of path planning has been mainly developed in robotics for motion planning and also navigation purposes [32].

Let us assume that X $\subseteq R^{d}$ (d ∈N, d ≥2) is a d-dimensional configuration space. By a configuration space we refer to a set whose members indicate all the states of an agent, a vehicle, a robot’s body or whatever that is supposed to be navigated [33]. Let X${}_{obs}$ and X${}_{free}$ also represent the obstacles and obstacle-free space, respectively, where: X${}_{free}$ = X ∖ X${}_{obs}$. Assuming that the initial condition x${}_{init}\in$ X${}_{free}$ and X${}_{goal}\subseteq$ X${}_{free}$, a path planning problem is defined as a triplet (X${}_{free}$, x${}_{init}$, X${}_{goal}$) [34], whose solution is a collision free path from x${}_{init}$ to X${}_{goal}$.

Path planning methods are in general categorized into 5 main groups including sampling based, node based optimal, mathematic model based, bio-inspired based and multi-fusion based algorithms [35]. In this work, due to the need to answer queries in as quick as possible in disaster situations, we have focused on the sampling based algorithms as they are on-line and quick in finding paths.

Sampling based algorithms and in particular RRT (Rapidly-exploring Random Tree) are based on a tree generation process whose nodes represent samples that are randomly selected from a given configuration space X. In our previous work, we developed on extension of RRT in order to involve the semantics of the map within the searching process [36]. After a brief explanation of the previous work, we will go into further details to see how we can better integrate the extended RRT with OntoCity and also the region retrieval process particularly for disaster scenarios where some regions (due to natural hazards) cannot be used by vehicles.

In RRT, the tree construction process starts by adding the first configuration point, x${}_{init}$, considered as the initial node of the tree, and continues till either a feasible path to the destination is found or the searching time ends [33]. The maximum searching time is set in the form of a parameter indicating the number of steps that the algorithm randomly fetches a configuration sample and adds it to the tree by linking it to the nearest node in the tree provided that the connection is collision free. To find a collision free path, these samples should necessarily belong to X${}_{free}$.

It is possible to end up with situations where no path is found due to the time limit set for a collision-free path searching process in a highly constrained environment [33]. For instance, in case of an Unmanned Aerial Vehicle (UAV), obstacles are understood as either elevation constraints or semantics constraints such as specific regions that should not be involved in any generated paths due to natural disasters such as flood.

Self-navigating vehicles such as drones are being increasingly used in outdoor environments for different purposes. What makes this process complicated and challenging for machines is the environment which is not always static. For instance, in case of disasters, some parts of the environment can be completely unreachable and therefore should be excluded from the X${}_{free}$ space. These changes in the environment increase the number of constrains and consequently the complexity of the path finding process.

In case of disaster, we extend RRT for improving its searching time using the semantics represented in OntoCity either in the form of path connectivity explained in Section 2.4 or the constraints considered in region retrieval process discussed in Section 4.1. The idea is to semantically enable RRT, or more specifically, to assist the path finding process by suggesting alternative regions for those which have changed and are not accessible anymore due to the catastrophe. It is worth mentioning that our typical application is about finding a way for a drone or a robot in disaster situations and not regular path planning scenarios as in logistics.

Given the populated OntoCity, let us assume that the system is asked to find a path between two regions without passing through/upon a specific region type C as a constraint. In order to find a path with such feature, after fetching a random sample ($x_{rand}$) from X${}_{free}$, RRT first checks to see if the random sample $x_{rand}$ belongs to the forbidden region C or not ( xrand?∈C). If it is the case ($x_{rand}\in C$), before extending the tree, RRT first runs the Query (21) explained in Section 2.4 (which is based on the path connectivity formed in OntoCity) to retrieve at least an alternative region for the forbidden region C. RRT then repeats the sampling process but this time within the newly retrieved region. In this way, we are just shifting all the randomly selected samples from forbidden regions to their alternatives that have similar behavior in terms of the connectivity of different regions (e.g., a sample taken from a *river* will be replaced by another sample that is instead located on a *bridge* around the *river*.) The rest of the RRT algorithm can be executed with no change.

One may pose the question: why before running the sampling process, we do not exclude all the forbidden regions from the configuration space. The answer is that depending on type, size, number and the geometry of constraints (i.e., forbidden zones) the region exclusion process can become highly time consuming. Moreover, the process of excluding regions from the configuration space would always be needed regardless of the location of the forbidden zones. However, in our suggested approach we apply this part of the reasoning only if an invalid sample is selected.

## 5. System Description

**Figure 8:**
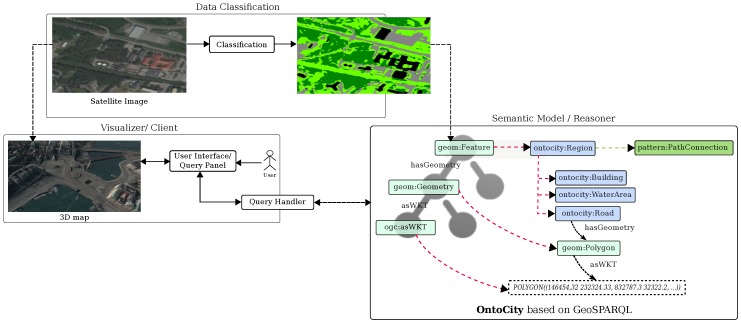
Semantic Representation and Reasoning as the main component of *SemCityMap*.

We have so far explained the full pipeline of the SemCityMap framework from data acquisition, to the enriching the raw image data with rudimentary labels, the integration of a knowledge representation and reasoning methods to enable high level querying. This pipeline with the relations among different components of the framework are represented in Figure 8. As we can see, OntoCity as the representation model accepts as input the classification results in the form of a set of labeled segments on the map. This representation together with the spatial connectivity of the instances of regions are able to be queried by the reasoner. The queries are generated by the users whom are provided an advanced graphical user interface (GUI) to express their needs according to the situation. The reasoner outputs which are either in the form of regions boundary or paths between regions are also represented upon the 3D map displayed by the GUI. Further details of the GUI are explained in the following subsection.

### 5.1. Advanced GUI

The user interface part of the system is developed as a standalone module, responsible for all interactions with the users, including forming map queries from user inputs, and results visualization. The secondary role of visualization provides a way to and performs visual inspection of the area of interest. Therefore, it serves a role of an efficient interactive tool for the user, is able to render large amounts of 3D data, performs queries, and displays information as overlays on top of the map.

The interface which is web-based is able to adjust the amount of 3D data to load from the server depending on required level of details based on current viewpoint and task.

The user interface is a browser-based application and is developed using HTML5/JavaScript, using WebGL1.0 specification. All 3D map data is served from a dedicated server in a form of files (in glTF format), representing tiles of the map at different levels of details. A tiling algorithm was implemented in order to minimize the amount of data to load and improve overall application performance. The tiling algorithm loads map tiles at the required levels of details based on camera elevation above sea level.

The interface also includes user interface controls to allow making map queries and visualizes the query responses as map overlays.

In order to allow better possibilities for visual inspection of the map, the visualizer uses smooth (Phong) shading, a realistic skydome rendering [37] depending on the current time/season at the point of interest, and animated water. The user interface allows adjusting date/time to simulate various lighting conditions and change level of water to visualize possible flooding scenarios.

Additionally to that, the visualizer allows map interaction using HMD-based devices (such as HTC Vive or Oculus Rift) by using WebVR browser specification in conjunction with a development (Nightly) version of Firefox web browser.

## 6. Results and Evaluation

### 6.1. Scenario

In order to investigate the performance of our semantically enabled region retrieval and path finding processes, we have defined a disaster scenario including rescue services. For the sake of simplicity, we have considered a simple drone as the self-navigating vehicle, with 1-dimensional configuration, where each single configuration is represented in the form of a 3D point in the space. In this case, the configuration space X is represented in 3D, where x, y and z coordinates are limited within a specific numeric range depending on the width, length and height size of the environment.

As mentioned in Section 1, the city that we used as example to illustrate our work is Stockholm in flood situation. The rescue team in a flood situation may receive different types of requests depending on the environmental conditions. For instance, one request might be finding safe regions around the flooded or dangerous areas (such as water area) where people may need help. The rescue team may also need to know the possible paths to the found regions which are only involving safe regions.

In the following subsection, we will show our results in data analysis, representation and reasoning and explain how using OntoCity and the semantically enabled RRT improves the searching time.

### 6.2. Results

**Figure 9:**
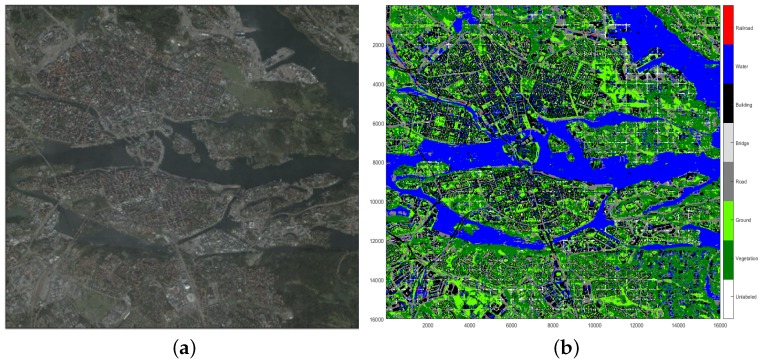
(**a**) Stockholm’s Satellite Data (as large as 64 km${}^{2}$); (**b**) Approximately 115,000 classified regions in Stockholm.

Available satellite data belongs to the central part of Stockholm, as large as 8 km × 8 km (see Figure 9a, which with 0.5 resolution results in a square shape area composed of 16,000 × 16,000 pixels. The 3D satellite imagery data of Stockholm along with its elevation data are provided by Vricon [38] as part of the Semantic Robot Project.

#### 6.2.1. Classification

Table 2 shows the confusion matrix for a classifier that has been trained on 90% of the initial labeled data (The details how we have gathered the initial manually labeled data can be found in [22]) with equal class distribution and tested on 1000 randomly drawn pixels per class from the remaining 10% of the labeled groundtruth.

#### 6.2.2. Population of OntoCity with Stockholm Satellite Data

Population of OntoCity includes separate processes of region, segment, event and path connectivity instantiation which are totally performed off-line (i.e., not at the query time). In the following, we describe the population process of the ontology with Stockholm’s satellite data.

The classifier applied upon the satellite data results in about 115,000 labeled regions shown in Figure 9b. However, before populating OntoCity with these regions, we first need to generate mutually disjoint segments (i.e., the instances of the ontocity:Segment class) that altogether cover all parts of the city.

Given the size of the map along with the number of classified regions, we found the size 1000 × 1000 pixels for each segment suitable w.r.t the computational complexity of the reasoning process. In this way, the map will be split into 256 (16 × 16) segments, where each will roughly contain between 410–470 regions that computationally sound a reasonable number of regions, particularly for geometrical/spatial calculations required in some queries.

Calculating the spatial relations between any pair of regions within a segment is also part of the ontology population process. On account of the segmentation  (Refers to the ontocity:Segment and not the segmentation process in the classification phase.) process upon the map, the time required for those geometrical calculations has been considerably improved. Each single geometrical calculation process takes on average 0.0014 s (The computer used has an Intel(R) Core(TM) vPro processor (2.60 GHz), 64 bit, 16 GB memory and Linux kernel 4.4.0-31-generic). The calculation time upon each segment is on average about 110 s which in total (for the entire map with 256 segments) takes about 29745 s (≈ 8 h).

The second process that is done off-line (i.e., not at the query time) is instantiation of classes that belong to the path connectivity pattern explained in Section 2.4. As we mentioned, the reason behind the connectivity pattern is to define a new affordance for some regions in terms of path connectivity. Reminding the example water area and bridges illustrated in Figure 4, we show how the instances of the classes in the path connectivity pattern contribute in enhancing the performance of the path finding process within a highly constrained environment. For instance, a region which is labeled as a *road*, connected to a water area, and at the same time located between at least two distinct shore areas (connecting them together), can be also seen or relabeled as a bridge in OntoCity. In order to find such regions we obviously need to run a query which holds all the aforementioned spatial criteria. For this, however, we have to first clarify what we mean by a shore area. As defined in Query (26), by shore, we refer to a ground area which is as such connected to (i.e., geos:touches) a water area.

(26) $$\begin{array}{cc} Shore\;=\;\{\;r_{i}\in R_{i}\;|\; & R_{i}\;⊑\;\mathrm{ontocity}:\mathrm{GroundArea}\;\wedge \\ & \;\exists \;r_{j}\in R_{j}\;⊑\;\mathrm{ontocity}:\mathrm{WaterArea}\;\wedge \\ & \;(r_{i},\;r_{j})\in S\;⊑\;\mathrm{geos}:\mathrm{touches}\;\} \end{array}$$

Given all the instances of the shore area, we eventually defined the relevant bridges as follows:

(27) $$\begin{array}{cc} Bridge\;=\;\{\;r_{i}\in R_{i}\;|\; & R_{i}\;⊑\;\mathrm{ontocity}:\mathrm{Road}\;\wedge \\ & \;\exists \;r_{j}\in R_{j}\;⊑\;\mathrm{ontocity}:\mathrm{WaterArea}\;\wedge \\ & \;(r_{i},\;r_{j})\in S\;⊑\;\mathrm{geos}:\mathrm{touches}\;\\ & \;\exists \;r_{s1}\in Shore\;\wedge \;\exists \;r_{s2}\in Shore\;\wedge \;r_{s1}\neq r_{s1}\;\wedge \\ & \;(r_{i},\;r_{s1})\in S\;⊑\;\mathrm{geos}:\mathrm{touches}\;\wedge \\ & \;(r_{i},\;r_{s2})\in S\;⊑\;\mathrm{geos}:\mathrm{touches}\;\} \end{array}$$

In this work, we have focused on the flood scenario and therefore, found it enough to only relabel those regions that can also be used as bridges over water areas. Perhaps, the same reasoning and query definition can be applied for other regions to get new labels useful in different situations for other scenarios. We have applied the relabeling process upon the central part of the map which is mainly surrounded with water (see Figure 9a) and therefore highly prone to further damages in case of flood. The central part of the map chosen for the relabeling process is composed of 42 (6 × 7) segments and totally includes about 18,000 regions. Given the two aforementioned queries, a single *road-to-bridge* relabeling process in a segment takes on average 0.8 s, which in total results in about 5 h for the selected area. It is worth mentioning that although by applying the query upon segments separately we may violate the completeness of the relabeling process (i.e., we may loose some regions adequate to be relabeled as bridges), however, we did so to save time at geometrical calculations (The exact relabeling time without segments has not been measured, but it will be more than 76 h for the selected area.). Geometrical calculations are necessary for determining the actual spatial relation (RCC-8) between two regions.

**Figure 10:**
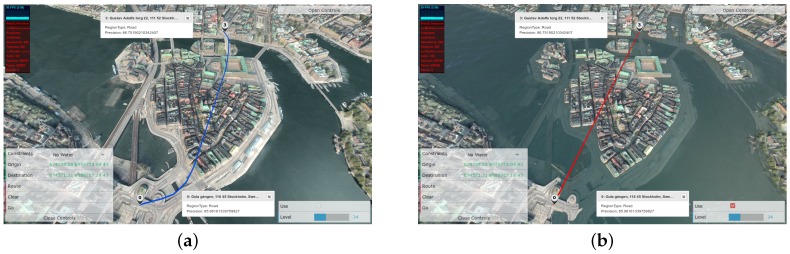
The same region of Stockholm in flooded and non-flooded conditions. The points 0 and 3 are the start and the destination points respectively. They are located on the different islands but there are possible paths between them through the bridges. (**a**) Non-flooded condition: the path finder is able to find a path with a “no water” constrain, using a bridge; (**b**) Flooded condition: the path finder is not able to find any path, because the destination point is located in the flooded area.

As mentioned above, another off-line process in OntoCity instantiation is about representation of events occurred in different areas. Instances of events in our work is generated by the simulator explained in Section 3.3.2. The simulator results in a number of polygons directly represented in OntoCity. In this work, we have flooded Stockholm at 73 different areas shown in Figure 10 with different sizes. As we can see in Figure 10a, the path finder finds a path using bridges if the constraint is “no-water areas”, which is not the case in a flood situation, as the bridges are also under water (see Figure 10b).

Given the populated ontology with regions, path connections and flooded areas, the users of the system (e.g., the rescue team) will be able to query the map according to their needs in confronting various environmental situations. For instance, a rescue team may need to find non-flooded regions around (between 100 to 1000 m) to a given flooded point on the map ($p_{f}$) in order to send a drone there and see if people get stuck there or not (see Query (28)). The query can be further extended and include spatial properties about these found regions. For instance, if it is needed to send a helicopter, these found regions should also hold another spatial feature saying that they are close or connected to at least one ground area with an area size big enough (e.g., 4 m${}^{2}$) for the helicopter to land (see Query (29)).

(28) $$\begin{array}{cc} \mathrm{Non}-\mathrm{Flooded}-\mathrm{Regions}\;=\;\{\;r_{i}\in R_{i}\;|\; & R_{i}\;⊑\;\mathrm{ontocity}:\mathrm{Region}\;\wedge \;R_{i}\;⋢\;\mathrm{ontocity}:\mathrm{WaterArea}\;\wedge \\ & \;100m\;\leq \;\mathrm{distance}(p_{f},\;r_{i})\;\leq \;1000m\;\wedge \\ & ∄\;a\in \mathrm{ontocity}:\mathrm{Area}\;\wedge \\ & \;(r_{i},\;a)\in \mathrm{ontocity}:\mathrm{intersects}\;\} \end{array}$$

(29) $$\begin{array}{cc} \mathrm{Non}-\mathrm{Flooded}-\mathrm{Regions}\;=\;\{\;r_{i}\in R_{i}\;|\; & R_{i}\;⊑\;\mathrm{ontocity}:\mathrm{Region}\;\wedge \;R_{i}\;⋢\;\mathrm{ontocity}:\mathrm{WaterArea}\;\wedge \\ & \;100m\;\leq \;\mathrm{distance}(p_{f},\;r_{i})\;\leq \;1000m\;\wedge \\ & ∄\;a\in \mathrm{ontocity}:\mathrm{Area}\;\wedge \\ & \;(r_{i},\;a)\in \mathrm{ontocity}:\mathrm{intersects}\;\wedge \\ & \;\exists \;r_{j}\in R_{j}\;⊑\;\mathrm{ontocity}:\mathrm{GroundArea}\;\wedge \;4m^{2}\;<\;size\left(r_{j}\right)\;\wedge \\ & \;(r_{i},\;r_{j})\in S\;⊑\;\mathrm{ontocity}:\mathrm{touches}\;\} \end{array}$$

#### 6.2.3. Query Time

In rescue scenarios, what matters is the response time of the queries. The main factor that influences the response time is the number of available regions. It means that we have to exclude regions as much as possible by applying a number of constraints that limit the number of candidate regions for the queries. One of the essential criterion is the distance intervals set in the query. The larger distance interval, the more regions to process. Although the distances are set by the users and we have no control on them, however, there are still other factors whose priority in the query execution process can considerably influences the response time.

We have considered 3 different setups for the same set of queries which along with their roles in reducing the query response time are shown in Table 3 and briefly explained in the following.

1. **Segmentation**: this factor allows us to first retrieve the relevant instances of the ontocity:Segment class (see Figure 7) to excludes all the regions belonging to all the non-relevant segments.
2. **Region Types Separation**: since in the queries we usually consider types of regions (e.g., water area, ground area, etc.) it can be helpful if we keep a separate list of regions for each segment (instead of a long list of regions regardless of their type), and only consider (or ignore) the regions with specific types mentioned in the queries.
3. **Flood Area Exclusion**: it obviously helps to first exclude the regions involved in the flood and then continue the geometrical/spatial calculations between regions.

For each criterion we have run 50 different queries to measure the average of the response time in both situations: with and without the criterion (see Table 3). Except for the segmentation factor which has been always considered, the inclusion/exclusion of the two other factors has been considered independent from each other to better study their roles. As we can see, applying each factor in the query causes the exclusion of some regions which consequently leads into shorter query time.

After retrieving the regions compatible with the desirable criteria, the rescue team may furthermore need to know the possible paths to the found regions which are only crossing safe regions.

**Figure 11:**
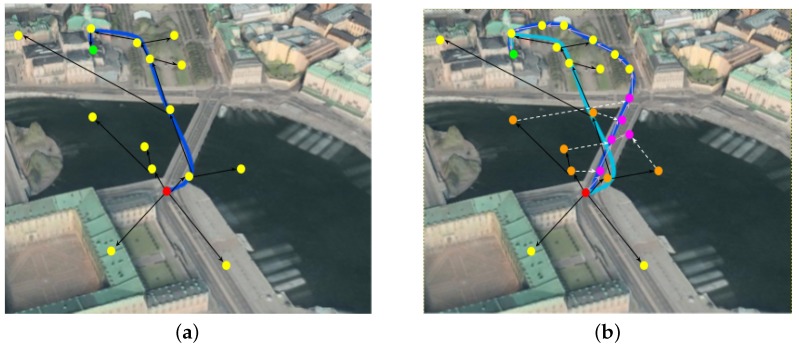
Path Finding With Constraints. The initial, goal and valid samples are in red, green and yellow, respectively. The generated collision-free path is in dark blue. (**a**) Constraint: Elevation of regions; (**b**) Constraint: Water Areas. Invalid samples are in orange and their alternatives are in pink.

Figure 11a shows a path that connects two points (x${}_{init}$ in red and x${}_{goal}$ in green) by crossing the river. Given water areas as forbidden zones, the path finding process would have difficulties in finding a path as the majority of samples are located in water areas due to their bigger area size. However, as depicted in Figure 11b, the path finding process replaces samples taken from the water area (x${}_{rand}$ in orange) with new samples taken from the bridge as an alternative (x${}_{alt}$ in pink) for the class ontocity:Water.

We have run the extension of RRT for 70 different path problems with different initial and goal points located in the central part of Stockholm. Table 4 shows the success rates with and without using the ontology reasoning during the tree construction process of the path finder. As we can see, the integration of semantics into the path generation process increases the success rates from 24.2% (17 successful cases out of 70) to 91% (64 successful cases out of 70) within 10 s set as the time limit for path finding. Without using the ontology, the average time for generating a path approaches the set upper limit of the planning process.

## 7. Conclusions

In this work we presented our framework designed to transform satellite imagery data into an interactive map ready to be queried. To achieve a queriable map directly from satellite data, a CNN classifier sensitive to the visual features (e.g., pixels’ color) of data has been applied to feed the framework with labeled regions. However, depending on the queries, the more advanced features of regions were needed to be taken into account. These features include pairwise spatial relations which together with regions’ texture also indicate their affordance in terms of path connectivity. Representing such features in an ontology which as such is an extension of the existing ontology GeoSPARQL, enables the system to automatically query the classified regions based on certain criteria of regions chosen based on the environmental situation.

We have shown that how by semantically enriching the representation of regions in OntoCity, we can enable the system to automatically find alternatives for regions, with an improvement in the time of queries including both region retrieval and path finding. The framework, SemCityMap, can now be used as tool to enable better decision support, and situational awareness. Using the city of Stockholm as an example, this paper has demonstrated the different functionalities that are available in SemCityMap.

## 8. Discussion and Future Work

It is worth noting that the work has also been validated on another smaller Swedish town of Boden [22], but future work will focus on several different improvements to the generic framework. First, errors in the preliminary CNN classification (misclassifications) could be addressed by leveraging from the integrated reasoner. In other words, when entities are misidentified (e.g., a shadow is misclassified as a road), we may want to exploit the high level domain knowledge in the ontology pattern to reason away these errors. Second, the reasoner can als be enhanced in terms of which type of agent is performing the query. For example, human operators may require that certain queries are answered in a manner that are adaptable to their needs. However, an autonomous vehicle, may require a different set of results. Our future work will examine how we can also take the constraints of the agent in the answering of the queries.

## Figures and Tables

**Table 1:** Spectral bands used in the multispectral orthography image.

Band	Bandwidth (nm)	Description
Red	630–690	Vegetation types, soils, and urban features
Green	510–580	Water, oil-spills, vegetation, and man-made features
Blue	450–510	Shadows, soil, vegetation, and man-made features
Yellow	585–625	Soils, sick foliage, hardwood, larch foliage
Coastal	400–450	Shallow waters, aerosols, dust, and smoke
Seafloor	400–580	Synthetic image band (Green, Blue, Coastal)
NIR1 (Near Infra-Red)	770–895	Plant health, shorelines, biomass, vegetation
NIR2	860–1040	Similar to NIR1
Pansharpened	450–800	High-resolution pan andlow-resolution multispectral
Soil	585–625, 705–745, 770–895	Synthetic image band (NIR1, Yellow, Red Edge)
Landcover	400–450, 585–625, 860–1040	Synthetic image band (NIR2, Yellow, Coastal)
Panchromatic	450–800	Blend of visible light into a grayscale
Red Edge	705–745	Vegetation changes
Vegetation	450–510, 510–580, 770–895	Synthetic image band (NIR1, Green, Blue)
DSM	-	Digital surface model

**Table 2:** Confusion matrix (%) for the 7 classes for a classifier trained and tested on the manually labeled data. The overall accuracy is 90.34%.

	Vegetation	Ground	Road	Bridge	Building	Water	Railroad
Vegetation	95.6	1.1	0.3	1.4	0.3	0.9	2.9
Ground	1.2	95.0	0.6	0.7	0.1	0.8	1.7
Road	0.2	1.3	94.1	3.4	0.1	0.4	3.8
Bridge	0.7	0.7	1.9	78.7	7.2	2.4	3.6
Building	0.2	0.1	0.1	9.2	91.0	0.4	0.9
Water	0.5	0.7	0.5	2.7	0.6	93.3	2.4
Railroad	1.5	1.1	2.5	3.8	0.6	1.9	84.7

**Table 3:** The average of the query response time with and without the criteria mentioned in the query. Each average time value has been calculated over running 50 different queries.

	Segmentation	Region Types Separation	Flood Area Exclusion
query time with	3 s	1.34 s	1.69 s
query time without	67 s	13.52 s	2.71 s

**Table 4:** Path Finder Performance.

	Without Ontology Pattern	With Ontology Pattern
Success Rate	24.2%	91%
Average of execution time	8.4 s	0.68 s

## References

[1] (2004). Climate Researchers Warn of Stockholm Floods. https://www.thelocal.se/20041204/706.

[2] Siricharoen W.V., Pakdeetrakulwong U. A survey on ontology-driven geographic information systems. Proceedings of the Fourth International Conference on Digital Information and Communication Technology and its Applicationsm DICTAP.

[3] Bekke J. (1992). Semantic Data Modeling.

[4] Alirezaie M. (2015). Bridging the Semantic Gap between Sensor Data and Ontological Knowledge. Ph.D. Thesis.

[5] Gangemi V.P.A., Staab R.S.S. (2009). Ontology Design Patterns. Handbook of Ontologies.

[6] Bittner T.M., Donnelly S.W. (2005). Ontology and semantic interoperability. Large-Scale 3D Data Integration: Challenges and Opportunities.

[7] Gu H., Li H., Yan L., Liu Z., Blaschke T., Soergel U. (2017). An Object-Based Semantic Classification Method for High Resolution Remote Sensing Imagery Using Ontology. Remote Sens..

[8] Almendros-Jimenez J.M., Domene L., Piedra-Fernandez J.A. (2013). A Framework for Ocean Satellite Image Classification Based on Ontologies. IEEE J. Sel. Top. Appl. Earth Obs. Remote Sens..

[9] Gao Y., Liu L., Lin X., Liu Y. (2013). A Qualitative Representation and Similarity Measurement Method in Geographic Information Retrieval. CoRR.

[10] Wang W., Dong C., Yang P. Ontology modeling of emergency plan systems. Proceedings of the 6th International Conference on Fuzzy Systems and Knowledge Discovery (FSKD).

[11] Huang W.D., Ding B.L., Yan L. (2013). The Design of Dynamic Response System Based on Digital Emergency Plan. Advanced Materials Research.

[12] Kalabokidis K., Athanasis N., Vaitis M. (2011). OntoFire: An ontology-based geo-portal for wildfires. Nat. Hazards Earth Syst. Sci..

[13] Agresta A., Fattoruso G., Pollino M., Pasanisi F., Tebano C., Vito S.D., Francia G.D., Murgante B., Misra S., Rocha A.M.A.C., Torre C.M., Rocha J.G., Falcão M.I., Taniar D., Apduhan B.O., Gervasi O. (2014). An Ontology Framework for Flooding Forecasting. Lecture Notes in Computer Science.

[14] Mostafavi M.A., Bakillah M. (2012). Real Time Semantic Interoperability in Adhoc Networks of GeoSpatial Data Sources: Challenges, Achievements and Prespectives. ISPRS Ann. Photogramm. Remote Sens. Spat. Inf. Sci..

[15] Battle R., Kolas D. (2012). Enabling the Geospatial Semantic Web with Parliament and GeoSPARQL. Semant. Web.

[16] Baader F., Nutt W. (2003). Chapter Basic Description Logics. The Description Logic Handbook.

[17] Cohn A.G., Bennett B., Gooday J., Gotts N.M. (1997). Qualitative Spatial Representation and Reasoning with the Region Connection Calculus. GeoInformatica.

[18] Hoehndorf R. What is an Upper Level Ontology?. http://ontogenesis.knowledgeblog.org/740.

[19] (2017). Defining N-ary Relations on the Semantic Web. https://www.w3.org/TR/swbp-n-aryRelations/.

[20] Maria Poveda M.C.S. Ontology Design Pattern. http://ontologydesignpatterns.org/wiki/Submissions:Symmetric_n-ary_relationship.

[21] LeCun Y., Bottou L., Bengio Y., Haffner P. (1998). Gradient-based learning applied to document recognition. Proc. IEEE.

[22] Längkvist M., Kiselev A., Alirezaie M., Loutfi A. (2016). Classification and Segmentation of Satellite Orthoimagery Using Convolutional Neural Networks. Remote Sens..

[23] Achanta R., Shaji A., Smith K., Lucchi A., Fua P., Susstrunk S. (2012). SLIC superpixels compared to state-of-the-art superpixel methods. Pattern Anal. Mach. Intell. IEEE Trans..

[24] (2017). OpenStreetMap. http://www.openstreetmap.org/.

[25] Alirezaie M., Längkvist M., Kiselev A., Loutfi A. Open GeoSpatial Data as a Source of Ground Truth for Automated Labelling of Satellite Images. Proceedings of the Workshop on Spatial Data on the Web (SDW 2016) Co-Located with The 9th International Conference on Geographic Information Science (GIScience 2016).

[26] Gangemi A., Guarino N., Masolo C., Oltramari A., Schneider L. Sweetening ontologies with DOLCE. Proceedings of the 13th International Conference on Knowledge Engineering and Knowledge Management.

[27] Teh C.H., Chin R.T. (1989). On the Detection of Dominant Points on Digital Curves. IEEE Trans. Pattern Anal. Mach. Intell..

[28] Ramer U. (1972). An iterative procedure for the polygonal approximation of plane curves. Comput. Graph. Image Process..

[29] Douglas D.H., Peucker T.K. (2011). Algorithms for the Reduction of the Number of Points Required to Represent a Digitized Line or its Caricature. Classics in Cartography: Reflections on Influential Articles from Cartographica.

[30] Carroll J.J., Dickinson I., Dollin C., Reynolds D., Seaborne A., Wilkinson K. (2004). Jena: Implementing the Semantic Web Recommendations. Proceedings of the 13th International World Wide Web Conference on Alternate Track Papers & Posters (WWW Alt. ’04).

[31] Guttman A. (1984). R-trees: A Dynamic Index Structure for Spatial Searching. Proceedings of the 1984 ACM SIGMOD International Conference on Management of Data (SIGMOD ’84).

[32] Jaillet L., Cortés J., Siméon T. (2010). Sampling-Based Path Planning on Configuration-Space Costmaps. IEEE Trans. Robot..

[33] Lavalle S.M., Kuffner J.J. Rapidly-Exploring Random Trees: Progress and Prospects. http://citeseerx.ist.psu.edu/viewdoc/download;jsessionid=5981B6747F51611A5FD1E02C563FF0F7?doi=10.1.1.38.1387&rep=rep1&type=pdf.

[34] Karaman S., Frazzoli E. (2011). Sampling-Based Algorithms for Optimal Motion Planning. Int. J. Robit. Res..

[35] Yang L., Qi J., Song D., Xiao J., Han J., Xia Y. (2016). Survey of Robot 3D Path Planning Algorithms. J. Control Sci. Eng..

[36] Alirezaie M., Kiselev A., Klügl F., Längkvist M., Loutfi A. Exploiting Context and Semantics for UAV Path-Finding in an Urban Setting. http://ceur-ws.org/Vol-1935/paper-02.pdf.

[37] Preetham A.J., Shirley P., Smits B. A practical analytic model for daylight. Proceedings of the 26th Annual Conference on Computer Graphics and Interactive Techniques (SIGGRAPH ’99).

[38] (2017). Vricon, Homepage. http://www.vricon.com.

